# Comparison of wild rice (*Oryza longistaminata*) tissues identifies rhizome-specific bacterial and archaeal endophytic microbiomes communities and network structures

**DOI:** 10.1371/journal.pone.0246687

**Published:** 2021-02-08

**Authors:** Xiaojue Peng, Jian Xie, Wenzhuo Li, Hongwei Xie, Yaohui Cai, Xia Ding

**Affiliations:** 1 School of Life Sciences, Nanchang University, Nanchang, Jiangxi, China; 2 Jiangxi Provincial People’s Hospital, Nanchang University, Nanchang, Jiangxi, China; 3 Jiangxi Super-Rice Research and Development Center, Jiangxi Academy of Agricultural Sciences, Nanchang, Jiangxi, China; Institute for Sustainable Plant Protection, C.N.R., ITALY

## Abstract

Compared with root-associated habitats, little is known about the role of microbiota inside other rice organs, especially the rhizome of perennial wild rice, and this information may be of importance for agriculture. *Oryza longistaminata* is perennial wild rice with various agronomically valuable traits, including large biomass on poor soils, high nitrogen use efficiency, and resistance to insect pests and disease. Here, we compared the endophytic bacterial and archaeal communities and network structures of the rhizome to other compartments of *O*. *longistaminata* using 16S rRNA gene sequencing. Diverse microbiota and significant variation in community structure were identified among different compartments of *O*. *longistaminata*. The rhizome microbial community showed low taxonomic and phylogenetic diversity as well as the lowest network complexity among four compartments. Rhizomes exhibited less phylogenetic clustering than roots and leaves, but similar phylogenetic clustering with stems. *Streptococcus*, *Bacillus*, and *Methylobacteriaceae* were the major genera in the rhizome. ASVs belonging to the *Enhydrobacter*, YS2, and *Roseburia* are specifically present in the rhizome. The relative abundance of *Methylobacteriaceae* in the rhizome and stem was significantly higher than that in leaf and root. Noteworthy type II methanotrophs were observed across all compartments, including the dominant *Methylobacteriaceae*, which potentially benefits the host by facilitating CH_4_-dependent N_2_ fixation under nitrogen nutrient-poor conditions. Our data offers a robust knowledge of host and microbiome interactions across various compartments and lends guidelines to the investigation of adaptation mechanisms of *O*. *longistaminata* in nutrient-poor environments for biofertilizer development in agriculture.

## Introduction

In natural environments, plants share their habitats with diverse microbiota, such as bacteria, archaea, fungi, oomycetes and viruses [1–3]. Many plant microbiota have substantial beneficial effects on their plant host, including improved nutrient uptake, accelerated growth, resilience against pathogens, and tolerance to biotic and abiotic stresses [1, 4, 5]. Plant microbiota can be found on the exterior of plants (the rhizosphere or the phyllosphere) and the interior of plants (the endosphere) [6]. Plant endophytes are microorganisms that live within plant tissues without causing disease [7, 8]. Plant endophytes are particularly fascinating given their plant growth-promoting (PGP) abilities, such as nitrogen fixation, the production phytohormone, ACC deaminase, phosphate solubilization, siderophore secretion and pathogen suppression [9–12]. The precise identification of plant endophytes is necessary to determine their distribution and biofunctions in the host and their beneficial effects.

Rice (*Oryza sativa*) is the most economically important crop in the world. The genus *Oryza* consists of cultivated species, *O*. *sativa* and *O*. *glaberrima*, and more than 22 wild species [13, 14]. The wild rice genomes provide an important pool of useful genes for rice improvement [15, 16]. Red rice, *Oryza longistaminata*, is a perennial wild rice with strong rhizomes. Rhizomes are horizontal, underground plant stems and essential organs that enable the plant to rapidly growth at the beginning of the next growing season [8, 17]. *O*. *longistaminata* has been used as the model species for genetic and molecular dissection of rhizome development and in breeding efforts to transfer rhizome-related traits into annual rice species [18, 19]. Moreover, *O*. *longistaminata* contains various valuable traits, including long anther, large biomass on poor soils, high nitrogen use efficiency, and resistance to insect pests and disease [15]. It has been hypothesized that survival of *O*. *longistaminata* in the nutrient-poor environments is associated with its microbiota. Flooded rice paddy fields represent a large contributor to agricultural CH_4_ emissions via biogeochemical processes that are mediated by soil and plant microbial communities [20–22]. Paddy-rice microbiome paly important role in the biogeochemical processes of C, N, P, and Fe, and the rice rhizosphere is one of the most dynamic habitats for element cycling due to carbon (C) input from roots [23]. Nitrogen is one of the most important nutrients for rice growth. Assessment of the diversity and community compositions of microbiota of *O*. *longistaminata*, especially the rhizome microbiota, is a fascinating area of research.

Whether the rhizome endophytes are mores similar to stems or roots remains unknown. Despite the significance of rhizomes in red rice [17, 24], little is known about the endophytic community structure and function of the rhizome. Endophytic communities of perennial *O*. *longistaminata* in rhizome may be different than those in the other compartments, given the role of rhizomes in adaption to nutrient-poor environments and functional differentiation [24]. In this study, we sequenced the root, rhizome, stem, and leaf endophytic microbiota in *O*. *longistaminata* and bulk soil. We compared the microbial communities and network structures in different compartments to investigate rhizome specificity and identify microbiomes that appeared to play important roles in rhizome function. Our work provides a basis for potential agricultural improvement of rice by endophytic microbiota modification.

## Materials and methods

### Sample collection and processing

The site for rice experiment is located at Nanchang city, China (28°40′04′′N, 115°49′31′′E). In May, 60 germinated seeds of *Oryza longistaminata* were transplanted into well-mixed red soil with 5 seeds in each tub. Rice was watered properly with tap water to ensure plant health and keep soil under flooding conditions, and harvested in October.

The roots, rhizomes, stems, and leaves of 12 *Oryza longistaminata* individuals, and 12 bulk soil were chosen for sampling, giving a total of 60 samples. The bulk soil samples were collected from unplanted pots approximately 2 inches below the soil surface. The soil was manually shaken from the roots. 5 cm long stem and rhizome were cut from 5 cm away from the node. Fragments of the roots, rhizomes, stems and leaves were washed with sterile water and separated. Then, the rice samples were treated with ultrasound. Ethanol-sodium hypochlorite was used for plant surface sterilization [25–27]. All the samples were washed successively in 70% ethanol for 1 min and 0.3% sodium hypochlorite with 0.01% Tween 20 for 15 min to further clean living microorganisms from the surfaces and subsequently washed the rice in sterile water. Finally, the sterile filter paper was used to absorb extra moisture. Water used for the final wash was spread on the Luria Broth (LB) and potato dextrose agar (PDA) plates to examine the surface sterilization effect.

### Characterization of the root and rhizome phenotype

The plants, roots and rhizomes were photographed using a Nikon E995 digital camera. The roots and rhizomes of *O*. *longistaminata* were dissected and vacuum infiltered with 4% (v/v) paraformaldehyde in phosphate-buffered saline (pH 7.0) for 30 min, and then renewed with fresh paraformaldehyde solution and incubated overnight at 4°C. Then, the fixed samples were dehydrated in a graded ethanol series, and embedded in Paraplast Plus (Sigma-Aldrich, St. Louis, MO, USA). Subsequently, materials were sectioned into 10-lm-thick sections, and stained with 0.1% aniline blue. 6 sections of each root and rhizome were viewed and photographed with an Olympus BX51 microscope [28, 29].

### Soil chemical properties analysis

Soil organic carbon (SOC) was measured with a TOC analyzer (Analytikjena HT1300, Germany) after removing soil carbonates using 1M HCl [22]. Inorganic nitrogen (NH_4_^+^, NO_3_^-^) was extracted and measured using 2 M KCl and a discrete auto analyzer (Smartchem 200, Westco, France). Soil pH was measured on soil slurry at a 2.5:1 water: soil ratio using a glass electrode [30].

### DNA extraction

Plant tissues were fully ground into powder in a mortar with liquid nitrogen. Then DNA was extracted from rice samples and bulk soil using the PowerSoil DNA isolation kit (Mo Bio Laboratories) as per the manufacturer’s instructions [31, 32]. DNA was quantified with a Qubit Fluorometer by using the Qubit dsDNA BR Assay kit (Life technologies, USA) and the quality was assessed by running an aliquot on a 1% agarose gel.

### 16S rRNA library construction

We performed 16S rRNA gene amplification for archaea and bacteria. Barcoded primers targeting the variable V4 regions of 16S rRNA genes were used for amplification with universal primer pairs, 515F (GGACTACNVGGGTWTCTAAT) and 806R (GGACTACHVGGGTWTCTAAT) [21]. Both forward and reverse primers were tagged with an Illumina adapter, pad and linker sequences. PCR enrichment was performed in a 50-μL reaction containing 30ng template, fusion PCR primer and PCR master mix. PCR cycling conditions were as follows: 95°C for 3 minutes, 30 cycles of 95°C for 45 seconds, 56°C for 45 seconds, 72°C for 45 seconds and final extension for 10 minutes at 72°C for 10 minutes. The PCR products were purified using Agencourt AMPure XP beads and eluted in Elution buffer. Libraries were qualified using the Agilent Technologies 2100 bioanalyzer. The validated libraries were used for sequencing on the Illumina HiSeq 2500 platform (BGI, Shenzhen, China) following the standard pipelines of Illumina, and generating 2 × 250 bp paired-end reads.

### Bioinformatics processing and statistical analysis

Bioinformatic processing steps and statistical analyses were conducted using QIIME 1.9.1, QIIME 2, usearch 11 and R versions 3.5.1 [33–35]. All amplicon sequence variants (ASVs) identified as belonging to chloroplast and mitochondria were removed from the data set. Rarefaction curves were calculated using QIIME 2. Sample metadata were predicted with random forest classification and regression models in QIIME 2 [36]. Principal coordinate analyses were calculated using the pcoa() function from the R package Ape. Permutational MANOVA was carried out to use Vegan’s function Adonis() to measure effect size and significances on β-diversity [37]. CAP analysis was performed using the function capscale() from the R package Vegan [38]. Variance partitioning and significances for experimental factors were performed by running Vegan’s permutest() function over the CAP model using a maximum of 500 permutations. Co-occurrence networks were inferred based on the Spearman correlation matrix constructed with R using the igraph package [39]. The Sankey diagram was plot in the R gallery (imageGP). Potential ecological functions of microbiota based on 16s rRNA gene sequences were predicted with FAPROTAX [40].

### Ethical approval

This article does not contain any studies with human participants performed by any of the authors.

## Results

### Compartment-specific microbiome taxonomic community structure of the wild rice microbiota

**Fig 1:**
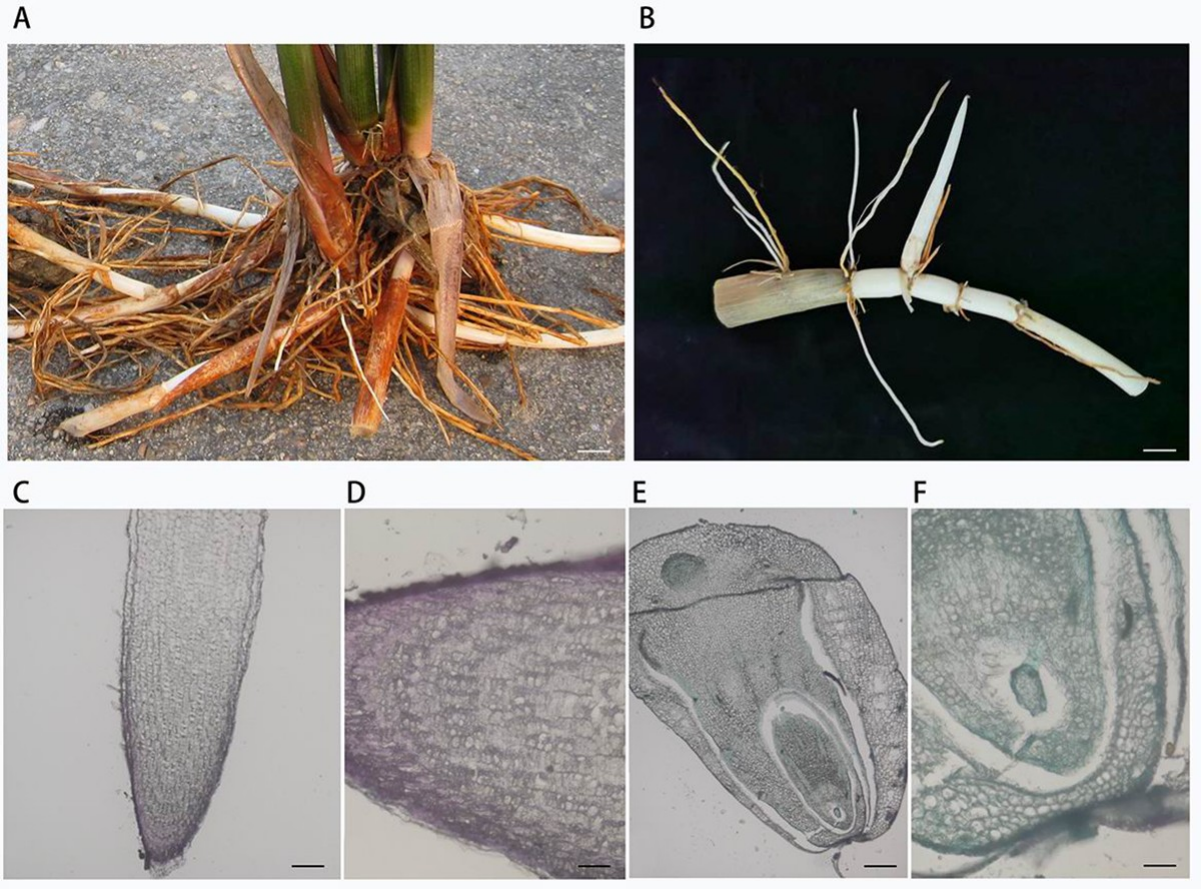
Wild rice (*Oryza longistaminata*) plant and rhizome. (A) Plants with strong rhizomes. Scale bar: 2 cm. (B) Rhizome and rhizome apical tips with sheath. Scale bar: 1 cm. (C-D) The vertical-section of the root apical tip. Scale bar: 160 and 40 μm. (E-F) The vertical-section of the rhizome apical tip. Scale bar: 160 and 40 μm.

The morphological characteristics of *O*. *longistaminata* showed strong rhizomes (Fig 1A and 1B), the anatomy structure of the rhizome was completely different from that of the root (Fig 1C–1F). We analyzed the bacterial and archaeal microbiomes from four separate compartments: the roots, rhizomes, stems, and leaves. For our study, the endophytic microbiomes, which are composed of the microbes inhabiting the interior of four compartments, were isolated from the four compartments after ultrasound-treatment and surface sterilization.

A total of 4,648,671 raw reads were identified among 48 samples of *O*. *longistaminata* (roots, rhizomes, stems, and leaves) and 12 bulk soil with a median read count per sample of 80,345 (range: 57,057–88,0877). Rarefaction curves were constructed for each sample showing that sufficient observations have been made (S1 Fig). Archaea were only detected in the bulk soil and root samples, and the relative abundance of archaea was significantly higher in soil than roots (S2A Fig). Notable differences in the proportions of various phyla are observed across the compartments (S3 Fig). At the phylum level, Proteobacteria (55.2%), Firmicutes (26.9%) and Bacteroidetes (7.0%) dominated microbial assemblages among the four compartments. A higher proportion of alpha-Proteobacteria (Kruskal-Wallis, p = 0.043) and Bacteroidetes (Kruskal-Wallis, p = 0.028) were observed in the aboveground parts (stems and leaves) than that in the belowground parts (roots and rhizomes), whereas the proportions of Firmicutes (wilcox, p = 5.24e-3) and Actinobacteria (wilcox, p = 1.12e-4) are higher in the belowground parts than the aerial parts (S3A Fig). *Rhizobium* (30.1%), *Streptococcus* (11.1%) and *Methylobacteriaceae* (7.4%) were the dominant genera in the leaves. *Methylobacteriaceae* (19.1%), *Neisseria* (11.4%) and *Staphylococcus* (10.0%) were the most prevalent genera in the stems. *Streptococcus* (27.7%), *Bacillus* (20.5%) and *Methylobacteriaceae* (15.3%) were the major genera in the rhizome. *Escherichia* (8.2%), *Treponema* (6.0%), *Pleomorphomonas* (5.3%), *Sulfurospirillum* (4.8%) and *Methylobacteriaceae* (2.0%) were the dominant genera in the roots (S3B Fig). This study reveals that the dominant *Methylobacteriaceae* was common in all four compartments of the wild rice, and the relative abundance of *Methylobacteriaceae* was 7.4%, 19.2%, 15.3% and 2.0% in leaves, stems, rhizomes and roots, respectively (S3B Fig). Other bacteria genera, including *Streptococcus* (7.8~27.7%), *Acinetobacter* (1.1~3.9%) and *Prevotella* (0.03~1.2%), were detected in all four niches.

**Fig 2:**
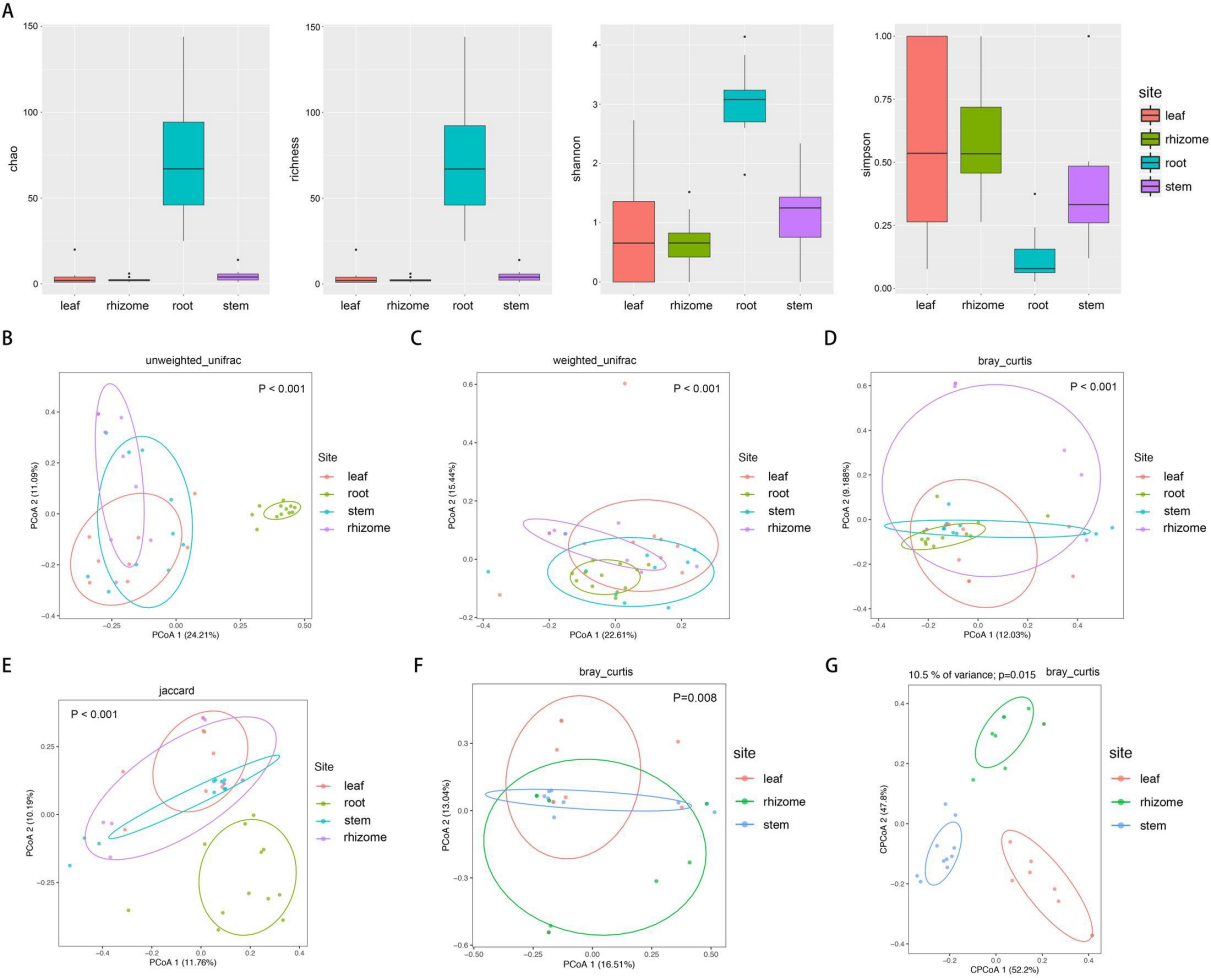
Microbiome alpha- and beta-diversity estimates. (A) Species richness estimates (Chao and richness) and diversity indices (Shannon and Simpson) for the 48 samples from roots, rhizomes, stems, and leaves of *O*. *longistaminata*. (B-E) Unconstrained principal coordinate analyses (PCoAs) of microbial community composition among four different compartments of *Oryza longistaminata* at the ASV level based on the unweighted UniFrac (UUF), weighted UniFrac (WUF), Bray-Curtis and Jaccard distance matrix. (F-G) PCoAs and CAP of microbial community composition among three different compartments of *Oryza longistaminata* at the ASV level based on the Bray-Curtis distance matrix.

The microbial alpha diversity within each sample was analyzed based on the species richness estimates (Chao1 and Richness) and diversity indices (Shannon and Simpson) (Fig 2A). Measures of alpha diversity revealed root communities had the highest α-diversity, and the α-diversity of roots was significantly higher than the other three compartments (rhizomes, stems and leaves) (Kruskal-Wallis test, P<0.01). However, the differences in α-diversity among the other three compartments (stems, rhizomes and leaves) were not be considered as statistically significant (Kruskal-Wallis test, p>0.05).

Unconstrained principal coordinate analyses (PCoAs) based on the unweighted UniFrac (UUF), weighted UniFrac (WUF), Bray-Curtis and Jaccard distance matrix were performed to investigate patterns of separation between microbial communities within different compartments. In the UUF and Jaccard PCoAs, the root compartments separate across the first and second principal coordinate respectively, indicating that the largest source of variation in microbial communities is proximity to the roots (PERMANOVA, P<0.001) (Fig 2B–2E). To more clearly observe patterns of separation between microbial communities within rhizomes, stems and leaves compartments, we performed PCoAs and partial canonical analysis of principal coordinates (CAP) based on Bray-Curtis distance matrix. PCoAs analysis indicated that microbial communities vary significantly between three compartments (PERMANOVA, P<0.01) (Fig 2F). We found that consistent with the PERMANOVA results, CAP analysis indicated that microbial communities were well separated among different compartments (P<0.05) (Fig 2G).

### Microbiome co-occurrence networks of ASVs in compartment-specific patterns

**Fig 3:**
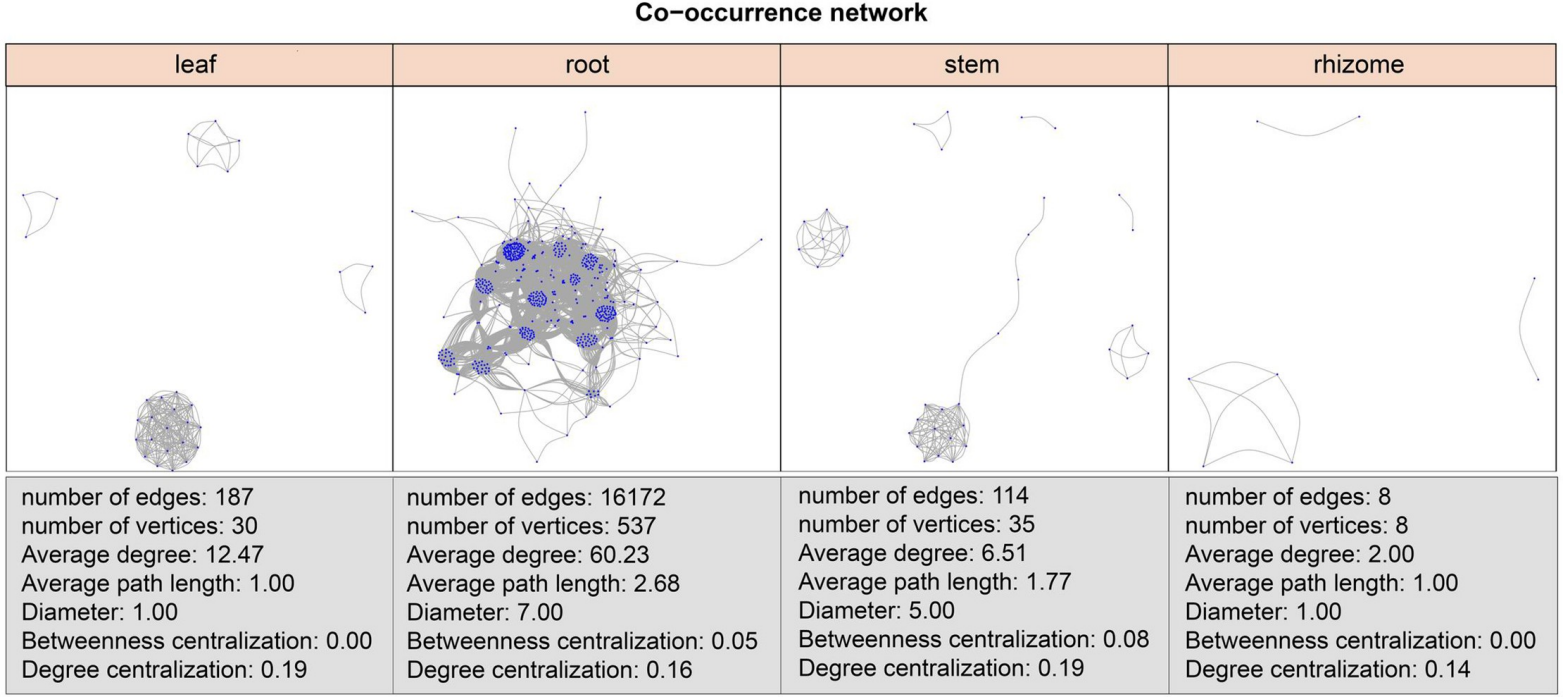
ASV co-occurrence network interactions of leaves, roots, stems and rhizomes. Co-occurrence relationships with strong Spearman’s correlation values (P-value < 0.5 and abs(r) > 0.6) are depicted for each compartment. The nodes represented unique ASV in the data sets.

We used significant correlations to construct co-occurrence networks, consisting only of positive correlations. Root co-occurrence networks were the largest capturing 16172 associations among 537 microbial ASVs with a higher average degree (60.23) (Fig 3). Rhizome co-occurrence networks were the smallest capturing 8 associations among 8 microbial ASVs with a lower average degree (2.00) (Fig 3).

### Classification and biomarker ASVs within different compartments

**Fig 4:**
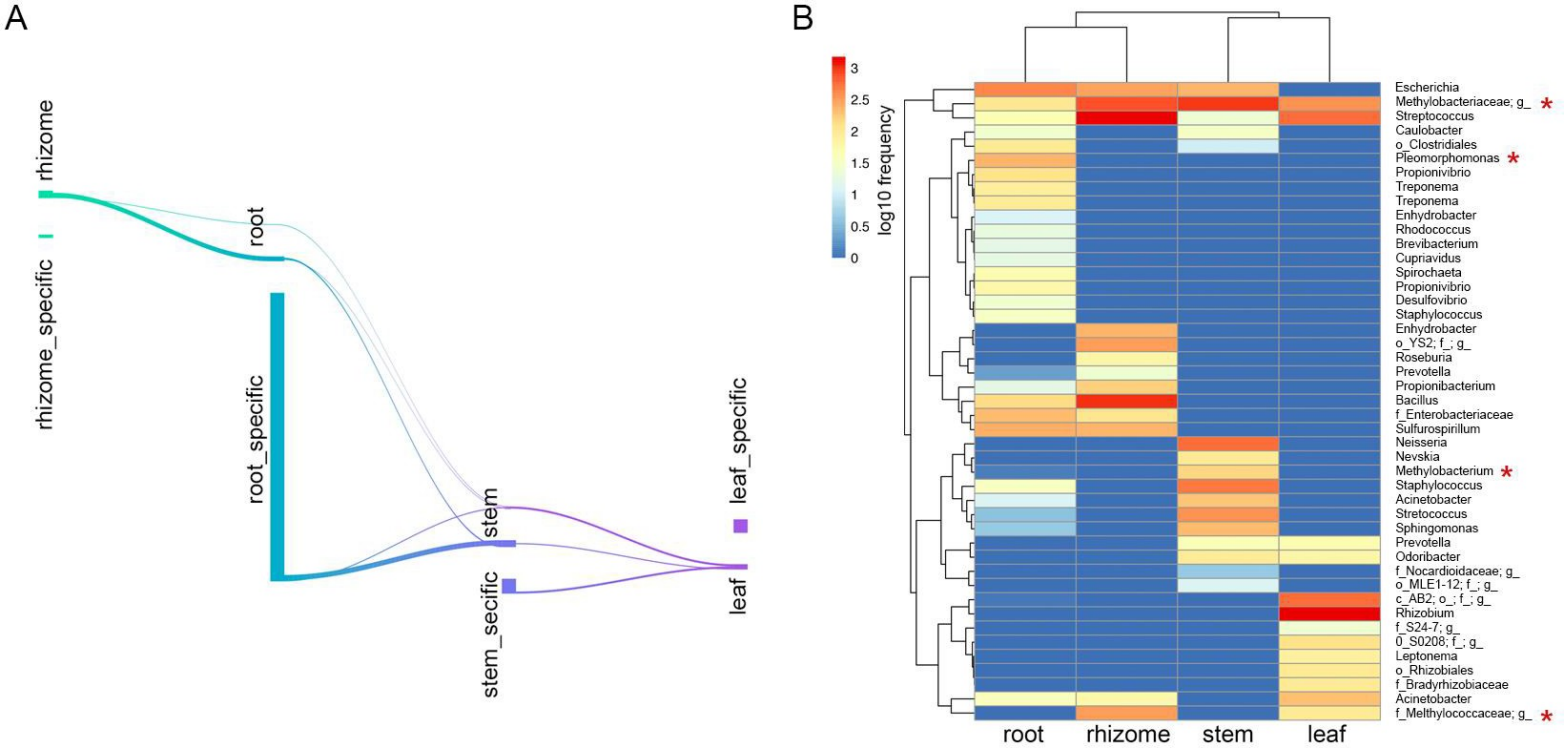
Distinctive taxa of the different compartments. (A) Numbers of differentially ASVs among each compartment in Sankey diagram. (B) Feature table heatmap of distinctive taxa in different compartments using random forest analysis. Methanotrophs are labeled by red stars.

To identify ASVs that correlated with the microbiome community in different compartments, we conducted differential ASV abundance analysis. Specific ASVs were identified in each compartment, and overlaps in differentially abundant ASVs was noted between the compartments (Fig 4A).

To gain insights into the biomarker ASVs of each compartment, we performed supervised learning with random forest methods to predict niche-specific ASVs. When the number of trees to grow for estimation was set to 500, the number of seeds used by the random number generator was set to 500, and the accuracy prediction was 75% (S4 Fig). ASVs belonging to the *Rhizobium*, AB2 and *Leptonema* were specifically presented in leaves. ASVs belonging to the *Neisseria*, *Methylobacterium* and *Nevskia* were specifically presented in stems. ASVs belonging to the *Enhydrobacter*, YS2 and *Roseburia* were specifically presented in rhizomes (Fig 4B). Some members within the *Methylobacteriaceae* and *Streptococcus* were common in all four compartments (Fig 4B). Noteworthy methanotrophs were observed across all compartments, including *Methylobacteriaceae*, *Pleomorphomonas*, *Methylobacterium*, and *Methylococcaceae* (Fig 4B).

### Phylogenetic community structure

**Fig 5:**
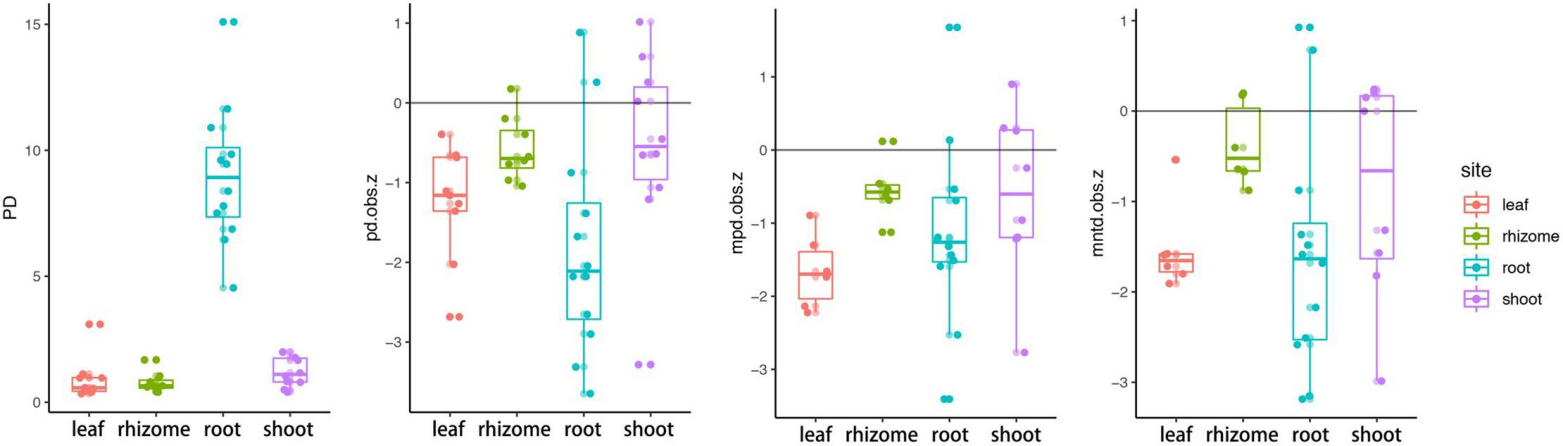
Phylogenetic community structure of *Oryza longistaminata*. PD, phylogenetic diversity; pd.obs.z, the standard effective size of phylogenetic diversity; mpd.obs.z, the standard effect size of mean pairwise distance among distinct taxa; mntd.obs.z, the standard effective size of mean nearest taxon distance among distinct taxa.

We computed phylogenetic diversity (PD) for each compartment. PD was highest for roots (Kruskal-Wallis, p = 1.007e-05), and the average PD of rhizomes was lower than that of stems, but there was no significant difference (Wilcoxon, p = 0.09) (Fig 5). The vast majority of ses.pd, ses.mpd, and ses.mntd were negative pointing to an increased tendency towards phylogenetic clustering. Leaf and root ASVs exhibited the more phylogenetic clustering than rhizome and stem, leaf and root had lower ses.pd (wilcox, p = 0.002), ses.mpd (wilcox, p = 0.007), and ses.mntd (wilcox, p = 0.014) than rhizome and stem, and the results presented high consistency among the three metrics.

### Microbes associated methane–nitrogen (CH_4_–N) cycle interactions

**Fig 6:**
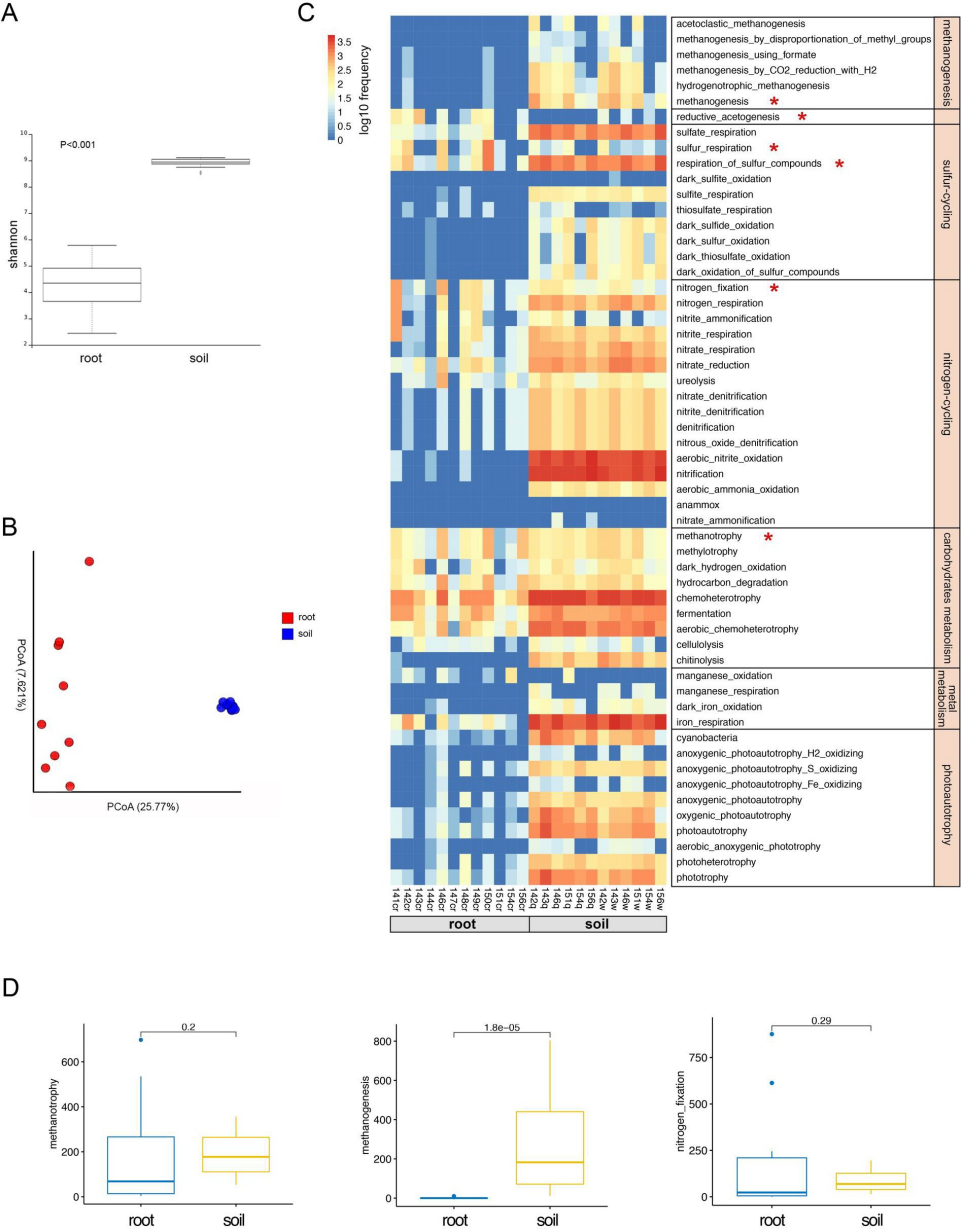
Microbiota diversity, community structure and functional analysis between the roots and bulk soil. (A) Alpha diversity estimates. (B) Beta diversity estimates. (C) ASVs involved in potential ecologically functions in roots and bulk soil. (D) ASVs involved in the potential CH_4_–N cycle between roots and bulk soil. Statistical significance was identified by the wilcoxon test with a false discovery rate (FDR)-corrected pairwise *P* values.

Root endophytic microbiota root communities exhibited the highest α-diversity and the most complex co-occurrence network compared with the other three compartments of *Oryza longistaminata* (Figs 2 and 3). It’s well known that soil microbiota plays a crucial role in biogeochemical processes and has a profound effect on soil functions. Using ASV counts from bulk soil as a control, we conducted a comparative analysis of root microbiota of *O*. *longistaminata* and paddy soil. The chemical properties of bulk soil was analyzed (S1 Table). The results showed that root communities had a lower α-diversity than soil (p<0.001) (Fig 6A), and the microbial community structure was significantly differed between roots and soil (PERMANOVA, P<0.001) (Fig 6B).

In order to compare the potential ecological functions between root and soil microbiota, we identified the potential biogeochemical functions of the endophytic microbiota of *O*. *longistaminata* using FAPROTAX. Our data showed that soil microbiota performed a complete set of biogeochemical processes, including methanogenesis, methanotrophy, nitrogen-cycling, sulfur-cycling, carbohydrates metabolism, metal metabolism and photoautotrophy (Fig 6C). However, root microbiota was involved in nitrogen-cycling, methanotrophy and sulfur-cycling (Fig 6C). Here we focused on the CH_4_–N cycle interactions. A large difference in methanogenic archaea was noted between the root and soil microbiota, the microbiome involved in methanogenic archaea in root was significantly lower than that of soil (Wilcoxon.test, p = 1.8e-5) (Fig 6D). Both root and soil microbiome had similar effect on the methanotrophy and nitrogen-fixation process (Wilcoxon.test, p>0.05) (Fig 6D).

## Discussion

Annually, irrigated rice production consumes approximately 10% of global fertilizer N and contributes to up to 19% of the global CH_4_ emissions [41]. How to reduce greenhouse gas emissions and N fertilizer use and improve rice yield has become an urgent problem that needs to be solved. Microbiota may help rice overcome these challenges. Although root-associated microbiota have been extensively studied, the role of microbiota living inside rice remains largely unexplored [42].

Plant genotype effects were significant on the plant microbiome [43–45]. Wild rice microbiota may contribute to the host’s adaptation to adverse environmental conditions. Thus, the study of the microbiota in wild rice is important for rice production and breeding. It has been reported that the rhizomicrobiota community structures differed between wild and cultivate rice [21, 46–49]. Here, we provide insights into the microbiota within the rhizomes, roots, stems and leaves of wild rice (*Oryza longistaminata*).

### Each compartment represents a distinctive ecological niche for endophytic microbiota

Annual and perennial habits are two different strategies by which rice adapt to seasonal environmental change [17, 50]. Rhizomes are a critical component of perenniality and allow wild rice to survive in a harsh environment [24]. Rhizomes are the original stem of plants, and plants rapidly regrow from rhizomes at the beginning of the next growing season [15]. Rhizomes are essentially underground modified stems. We are interested in the difference between the microbes of underground and aboveground stems. In addition, rhizomes and roots are underground tissues and have similar soil/rhizosphere environments. We would like to know if the microbial diversity and structure in the rhizomes are more similar to stems or roots. To answer this question, a comparison to the roots, rhizomes, stems, and leaves microbial assemblages was conducted.

Our data revealed enrichment of members of the phyla Proteobacteria, Firmicutes and Bacteroidetes and the genus *Methylobacteriaceae* in all four compartments of the wild rice (S2 Fig). In each of the four compartments, we detected marked “compartment-specific” structural and phylogenetic diversification. The rhizome microbiota is unique and possessed niche-specific microbial taxa (*Enhydrobacter*, YS2, and *Roseburia*) (Fig 4). Although rhizomes are underground tissues like roots, the microbial diversity and community network structures of the rhizomes were surprisingly significantly reduced compared to the roots (Figs 2, 3 and 5). Meanwhile, the microbial community of the rhizomes exhibited similar taxonomic and phylogenetic diversity compared with that of stems, and even lower network complexity compared with that of stems (Figs 2, 3 and 5). The data showed that the rhizome endophytic microbiota of *O*. *longistaminata* was significantly different from other tissues, especially the root, supporting the notion that endophytic microbiota of the rhizome are more similar to that of stems compared with that of roots. Ruifeng et al compared the transcriptome, proteome, and metabolome of the rhizome to other tissues of *O*. *longistaminata*, and found that metabolite profiling of the rhizomes was significantly different from the roots but similar with that of stems [15]. Taken together, we hypothesized that the rhizome endophytic microbiota was indeed more similar to that of stems compared with that of roots partly due to the metabolite characteristics of the rhizomes.

Compared with wild rice, Proteobacteria dominated the root endophyte community in cultivated rice (*O*. *sativa*) [41, 46]. In our data, the phyla Proteobacteria dominated all four compartments of *O*. *longistaminata*, which in agreement with cultivated rice (*O*. *sativa*). However, the microbial community structure of other wild rice reported in the literature was different from that of *O*. *longistaminata*. To investigate the relationship between rice genotype and the root microbiome, Joseph et al., compared Asian cultivated rice (*O*. *sativa japonica* and *indica* varieties) and African cultivated rice (*O*. *glaberrima*), the results demonstrated that microbial communities were influenced by rice genotype [21]. To compare of the root-associated microbiomes in wild (*O*. *rufipogon*) and cultivated rice varieties (*O*. *sativa* L. ssp. japonica), Lei et al., found that the relative abundance of *Streptomyces*, *Enterobacteriaceae*, *Leptolyngbya*, *Planctomycetes*, *Acidovorax*, *Ohtaekwangia*, *Planctomycetaceae*, and *Comamonadaceae* were higher in wild rice, the relative abundance of *Acidovorax*, *Azospirillum*, *Ohtaekwangia*, *Actinoplanes*, *Comamonadaceae*, *Oxalobacteraceae*, *Myxococcales*, and *Acidobacteria* were higher in cultivated rice [47]. Recently, Veronica et al., profiled the leaf microbial community composition across 3024 rice (*O*. *sativa*), the result showed that *Proteobacteria*, *Firmicutes*, *Actinobacteria*, *Cyanobacteria*, *Tenericutes*, and *Euryarchaeota* were the most abundant phyla [51].

### Symbiotic type II methanotrophs may represent key microbes for *Oryza longistaminata* survival under the nutrient-poor condition

The relationships between biogeochemical processes and microbial functions in paddies have received considerable. Metagenomic analysis showed that root endophytes of cultivated rice (*O*. *sativa*) might be involved in the entire nitrogen cycle, as N_2_-fixation, denitrification, and nitrification [41, 52]. Our data suggested that root microbiota are involved in the nitrogen cycling, methanotrophy and sulfur cycling (Fig 6). Soil microbiota performed a complete set of biogeochemical processes, including methanogenesis, sulfur cycling, nitrogen cycling, carbohydrate metabolism, metal metabolism and photoautotrophy (Fig 6). Methane is mostly synthesized by methanogenic archaea in paddies. We found a large difference in the abundance of methanogenic archaea between the rice paddy soil and roots. Methanogenic archaea exhibit increased higher ASVs relative abundance in the soil compared with the roots (Wilcoxon, p = 2.234e-5) (S2 Fig). The function prediction confirms the ASV relative abundance results, suggesting that soil microbiota but not root microbiota are involved in methanogenesis (Fig 6C). Under flooded conditions, paddy soil exhibits anoxic conditions [53]. Oxygen is subsequently transported from the atmosphere into roots; thus, rice roots grow under partially oxic conditions [54, 55]. Methanogenic archaea require anoxic conditions; thus, we observed that methanogens inhabit flooded rice soil, but not in rice roots (S2 Fig).

CH_4_ produced from the anoxic zone of paddy soils by methanogenic archaea is transported into the rice via the aerenchyma tissues in the rhizomes, roots and stems [56, 57]. The rice tissues provide a microenvironment full of inorganic and organic substances, including C1 compounds such as methane, which allows the growth of methanotrophy bacteria that utilize CH_4_ and methanol as carbon sources [20, 58]. Thus, a considerable number of methanotrophs are present in the rice tissues due to adequate CH_4_ supply from paddies (Figs 4 and 6). Previous studies reported that type I methanotrophs were enriched in cultivated rice (*Oryza sativa*) roots compared to type II methanotrophs [54]. Moreover, type II methanotrophs in paddies dominate in environments with high concentrations of CH_4_ but limited nitrogen compared with the type I methanotrophs possibly due to the ability of type II methanotrophs to mediate CH_4_ oxidation and fix nitrogen (N_2_) [20, 59]. Nitrogen is one of the most important nutrients for rice growth. It was found that type II methanotrophs are the predominant root-associated microbiota in the rice roots with no N input [52, 58, 60]. *Oryza longistaminata* broadly distributed throughout tropical Africa exhibits high biomass production on poor soil [17]. In the study, noteworthy methanotrophs were identified across all compartments, including *Methylobacteriaceae*, *Pleomorphomonas*, *Methylobacterium*, and *Methylococcaceae* (Fig 4B). Most of these methanotrophs belong to type II methanotrophs [54, 61, 62]. Taken together, we may hypothesize that the wild rice (*Oryza longistaminata*) exhibits the potential for CH_4_-dependent N_2_ fixation by type II methanotrophs as a mechanism to adapt the CH_4_-enriched environment under nitrogen nutrient-poor conditions. The ecological function of this mechanism requires further research.

## Conclusions

The phytobiome plays a crucial role in plant nutrition and fitness. Wild rice is an important model to study plant-microbe interactions given their adaption to arid and infertile environments. Here, diverse diversity microbiota and significant variation of community structure were observed among different compartments of *O*. *longistaminata*. Rhizome, a critical component of perenniality, harbored specific microbiomes. Rhizome endophytic microbiota of *O*. *longistaminata* are significantly different from other tissues, especially the root, supporting the notion that endophytic microbiota of the rhizome are more similar to that of stems compared with that of roots. We found that the relative abundance of *Methylobacteriaceae* in rhizome and stem is significantly high. The dominant microbiome of *O*. *longistaminata*, type II methanotrophs, may benefit the host by facilitating CH_4_-dependent N_2_ fixation under nitrogen nutrient-poor conditions. Overall, this study provides an opportunity to understand plant-microbiome interactions in different compartments of red rice.

## Supporting information

S1 Fig Microbial community composition at different taxonomic ranks. The distributions of microbial phylum (A) and genus (B) at different compartments of *Oryza longistaminata*.
(TIF)

S2 Fig Rarefaction curves of all the samples of *Oryza longistaminata* revealed by 16S rRNA. (A) Observed ASVs. (B) Shannon index.
(TIF)

S3 Fig Classification accuracy results of supervised learning classifiers. (A) Model accuracy. (B) Receiver operating characteristic curves.
(TIF)

S4 Fig Microbial community composition at different taxonomic ranks. The distributions of the microbial domain (A) and phylum (B) in roots and bulk soil.
(TIF)

S1 Table Soil physicochemical data. (XLSX)
