# Supplementary figures and images for: Comparison of wild rice (Oryza longistaminata) tissues identifies rhizome-specific bacterial and archaeal endophytic microbiomes communities and network structures
Source: PLoS One. 2021 Feb 8;16(2):e0246687. doi: 10.1371/journal.pone.0246687 (PMC7870070; doi:10.1371/journal.pone.0246687)

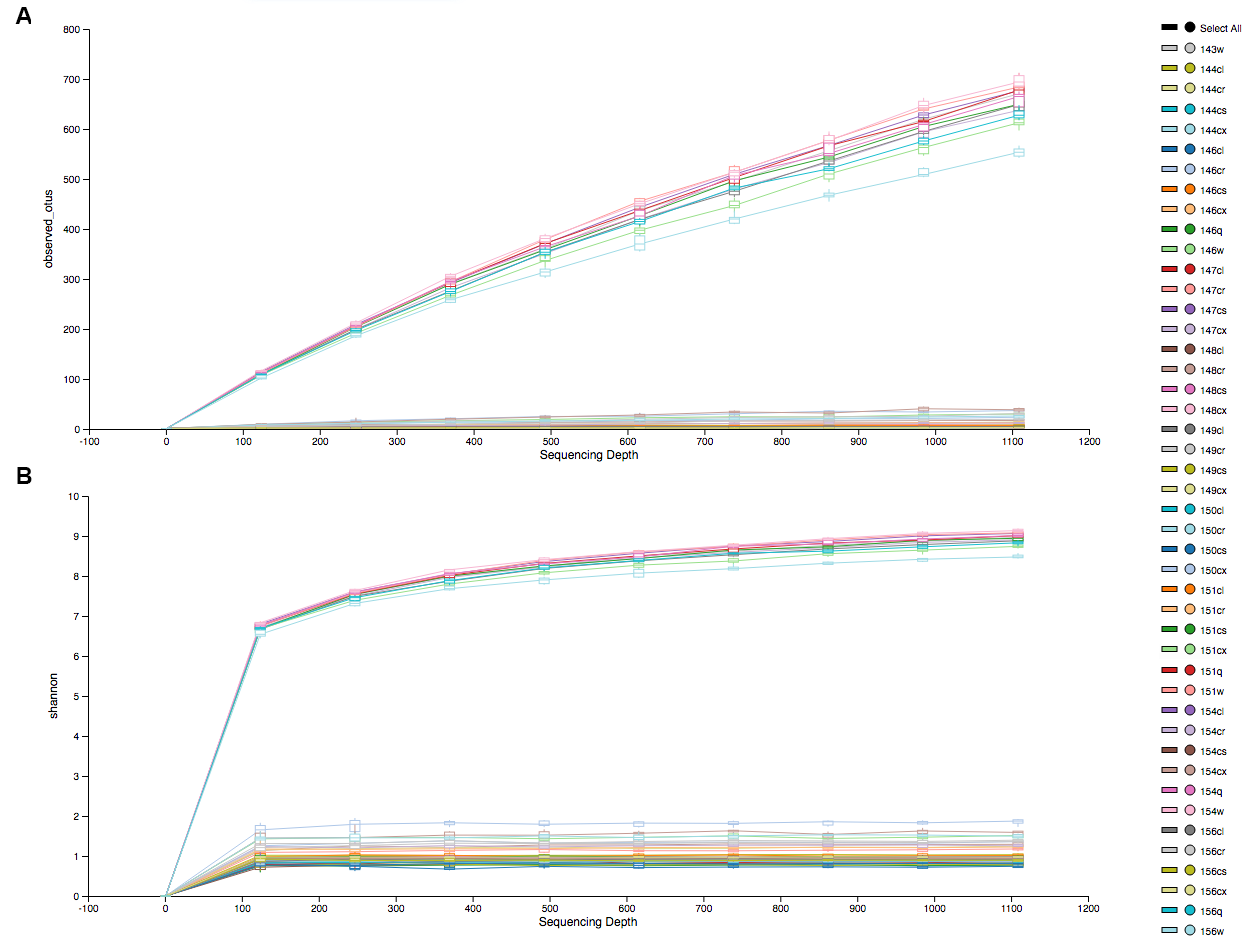

Supplement: S1 Fig — The distributions of microbial phylum (A) and genus (B) at different compartments of Oryza longistaminata. (TIF) [file pone.0246687.s001.tif]

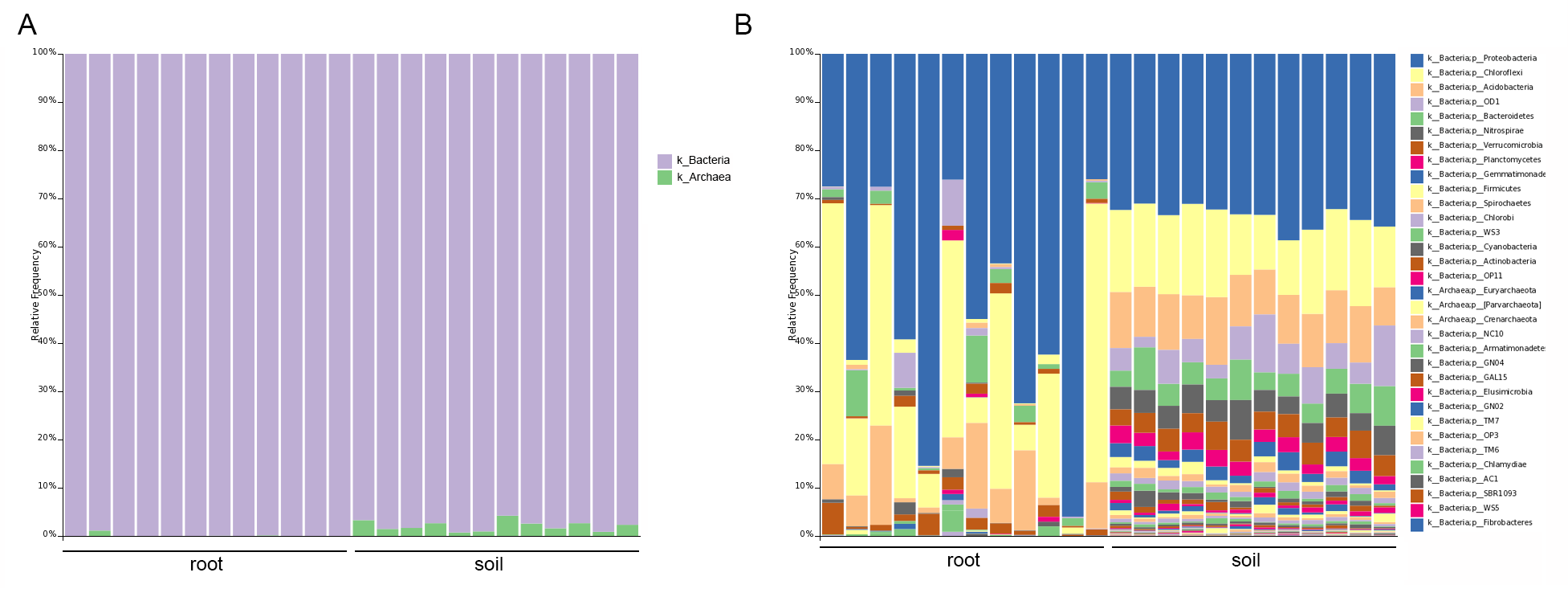

Supplement: S2 Fig — (A) Observed ASVs. (B) Shannon index. (TIF) [file pone.0246687.s002.tif]

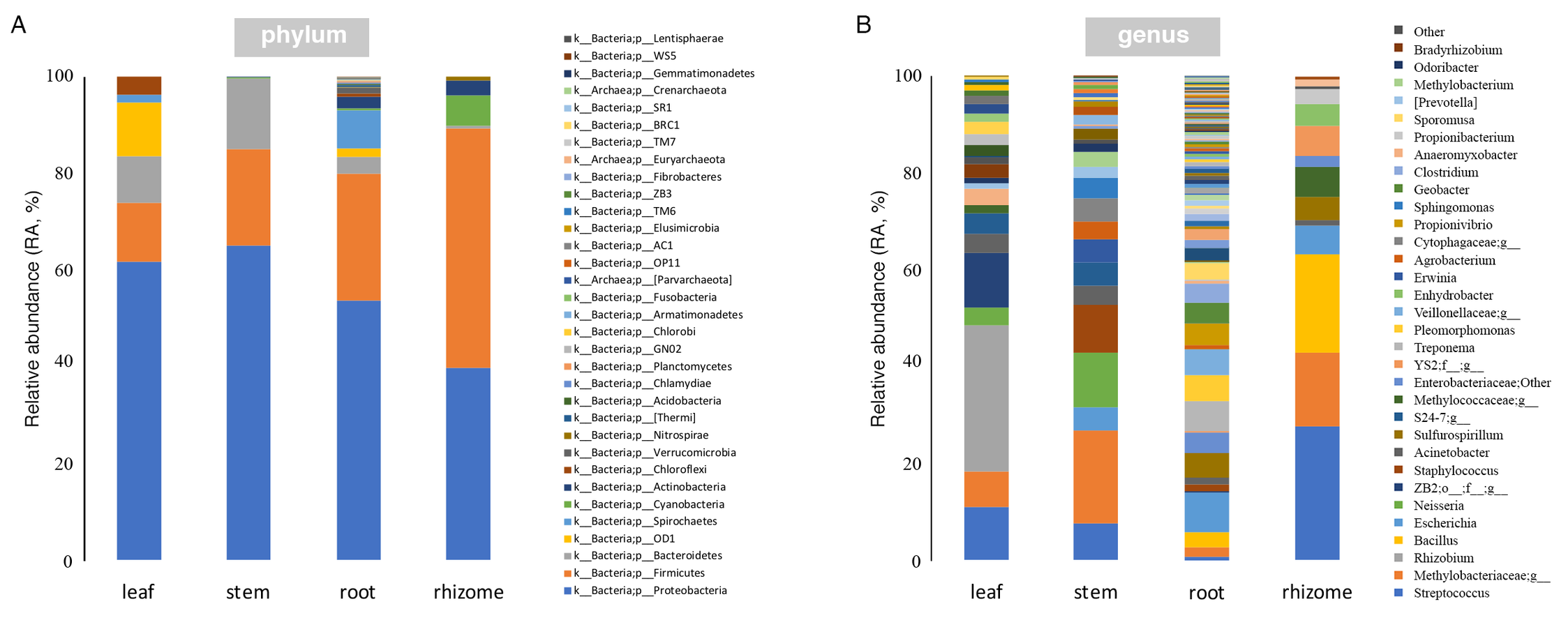

Supplement: S3 Fig — (A) Model accuracy. (B) Receiver operating characteristic curves. (TIF) [file pone.0246687.s003.tif]

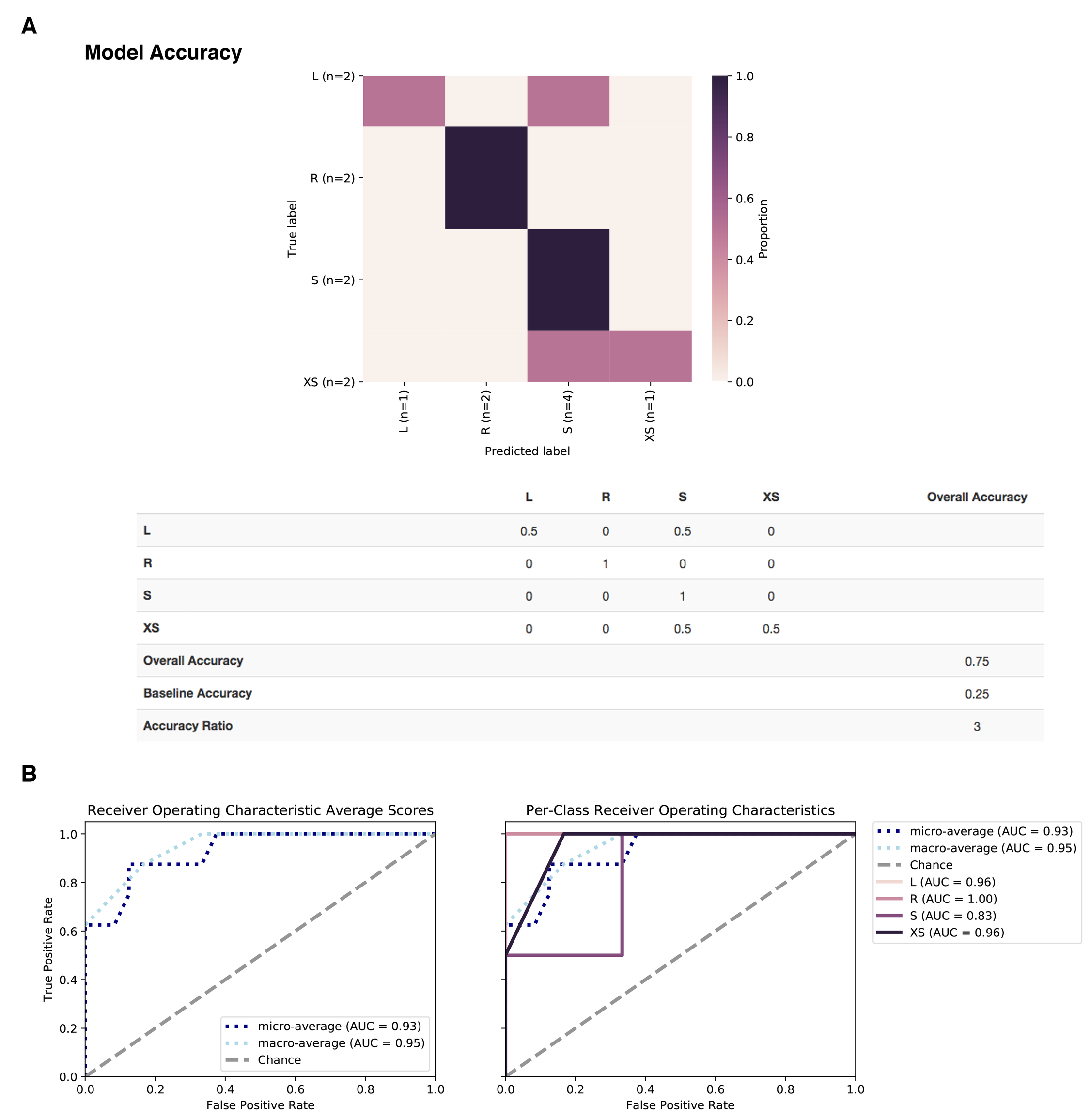

Supplement: S4 Fig — The distributions of the microbial domain (A) and phylum (B) in roots and bulk soil. (TIF) [file pone.0246687.s004.tif]
